# Clinical efficacy of selenium supplementation in patients with Hashimoto thyroiditis: A systematic review and meta-analysis

**DOI:** 10.1097/MD.0000000000044043

**Published:** 2025-08-29

**Authors:** Heng Zhang, Yunkai Yang, Shaohua Liu, Yang Yang, Zhelong Liu

**Affiliations:** a Department of Endocrinology and Metabolism, General Hospital of The Yangtze River Shipping, Wuhan Brain Hospital, Wuhan, China; b Eight-year Program of Clinical Medicine, Tongji Hospital, Tongji Medical College, Huazhong University of Science and Technology, Wuhan, China; c Department of Endocrinology, Tongji Hospital, Tongji Medical College, Huazhong University of Science and Technology, Wuhan, China; d Department of Endocrinology, Tianyou Hospital Affiliated to Wuhan University of Science and Technology, Wuhan, China.

**Keywords:** Hashimoto thyroiditis, levothyroxine, meta-analysis, selenium supplementation, selenomethionine

## Abstract

**Background:**

To assess the clinical benefits of Selenium (Se) supplementation in patients with Hashimoto thyroiditis (HT).

**Methods:**

Eight databases were searched for randomized controlled trials. The outcomes of interest were thyroid peroxidase antibody (TPOAb), thyroglobulin antibody (TgAb), thyroid-stimulating hormone (TSH), free triiodothyronine (FT_3_), and free thyroxine (FT_4_). The study protocol is registered on INPLASY, DOI number is 10.37766/inplasy2022.10.0085.

**Results:**

Twenty-one studies with a total of 1610 subjects were included. Serum TPOAb was significantly reduced after Se supplementation after 3 months (standardized mean difference [SMD] = −0.46, 95% confidence interval [CI]: −0.74 to −0.18, *P* = .001) and 6 months (SMD = −0.80, 95%CI: −1.38 to −0.21, *P* = .008). The serum TgAb levels decreased at 3 months (SMD = −0.46, 95%CI: −0.79 to −0.12, *P* = .007) but not at 6 months. Significant effects on declining the TSH titers were found after 6 months (SMD = −0.18, 95%CI = −0.35 to −0.01; *P* = .03).

**Conclusion:**

Se supplementation help reduce TPOAb and TSH levels in HT patients, leading to improvements in well-being or mood. Selenomethionine is more effective than NaSe and Se-yeast in the teatment of Hashimoto thyroid.

## 1. Introduction

Hashimoto thyroiditis (HT) is a prevalent autoimmune disease and the most common endocrine disorder,^[1,2]^ accounting for the majority of cases of hypothyroidism.^[3,4]^ Despite being first described over a century ago, the exact cause of HT is not yet fully understood.^[5]^ One of the hallmarks of the disease is the infiltration of T lymphocytes into the thyroid gland, leading to goiter formation. Clinical diagnosis often includes elevated levels of antithyroglobulin antibody (TgAb) and antithyroid peroxidase antibody (TPOAb).^[6,7]^ HT affects about 1% to 2% of the general population, with a higher prevalence in women and an increasing incidence with age.^[8,9]^ Low levels of thyroid hormones are primarily responsible for the clinical manifestations of the disease, and hormone replacement therapy, such as levothyroxine (LT_4_), is the standard treatment.^[8–10]^ The etiology of HT is multifactorial, involving genetic predisposition and environmental triggers, with selenium (Se) deficiency being a potential contributing factor.^[11]^

Se is an essential trace element for the human body, and its deficiency has been associated with the development of autoimmune diseases, including HT. However, the exact role of Se in HT pathogenesis remains unclear, and further research is needed to establish the link between the 2. The thyroid gland contains the highest concentration of Se per unit of tissue in the body^[12]^ indicating the essential role of this trace element in thyroid function. Se is primarily stored in the body as selenoprotein that plays a crucial role in the immune system and activation of thyroid hormones.^[13,14]^ In patients with autoimmune thyroid diseases, the Se levels are significantly low, leading to weakened resistance against oxidative stress.^[15]^ The synthesis of thyroid hormone requires the enzyme thyroid peroxidase (TPO) to convert iodine into active iodine, which then iodinates tyrosine residues under the oxidation of hydrogen peroxide (H_2_O_2_). However, this process produces excess H_2_O_2_, the accumulation of H_2_O_2_ and reactive oxygen intermediates results in the destruction of thyroid cells, causing the release of thyroglobulin and TPO into the bloodstream and triggering autoimmune reactions. Among patients with HT, those with Se deficiency tend to have higher levels of thyroid-stimulating hormone (TSH), TPOAb and TgAb compared to patients who are selenium-sufficient.^[16]^ Selenoenzymes, including glutathione peroxidases (GPx), TR, iodothyronine deiodinases, and selenoprotein P, play a critical role in human thyroid function and thyroid hormone homeostasis among at least 30 selenoproteins.^[17,18]^ GPx, a selenoprotein with strong antioxidant activity, can remove excess hydrogen peroxide, peroxides, and oxygen free radicals in the thyroid gland, maintaining cell membrane integrity and affecting thyroid hormone synthesis.^[19]^ Patients with HT who take Se supplements exhibit altered inflammatory and immunological responses due to increased plasma GPx and TR activity, as well as decreased harmful levels of H_2_O_2_ and lipid hydroperoxides produced during thyroid hormone production.^[17,20]^ A meta-analysis^[21]^ of 4 original studies^[22–25]^ published in 2010 demonstrated that Se supplementation for 3 months led to a significant decrease in TPOAb levels and improved well-being or mood compared to placebo. Similar conclusions were also observed in other literature.^[26]^ However, some studies did not show a significant decrease in TPOAb titers upon Se supplementation, suggesting that the clinical significance of TPOAb in the treatment of HT patients remains uncertain.^[27]^

The use of Se supplementation as a potential treatment for HT has been the subject of much debate among medical professionals, with conflicting evidence regarding its efficacy. This meta-analysis aims to evaluate the available data to provide an evidence-based recommendation regarding the use of Se supplementation in the management of HT.

## 2. Methods

This meta-analysis of randomized controlled trials (RCTs) was performed following the Preferred Reporting Items for Systematic Reviews and Meta-Analyses (PRISMA)^[28]^ statement and was registered in INPLASY (registration number is INPLASY2022100085, DOI number is 10.37766/inplasy2022.10.0085).

### 2.1. Search strategy

We conducted a comprehensive search of several databases including CNKI, WANFANG, VIP, CBM, PubMed, Web of Science, Embase, and Cochrane Library for RCTs on Se and HT published from the inception of each database to April 4, 2022. The search was performed using a combination of subject headings and free terms in accordance with the Cochrane Collaboration Handbook. Chinese search terms included “thyroiditis, autoimmune,” “chronic lymphocytic thyroiditis,” “thyroiditis, lymphoma,” “Hashimoto thyroiditis,” “autoimmune thyroiditis,” “Hashimoto thyroiditis,” “lymphoid thyroiditis,” “selenium,” “selenium yeast,” “selenite,” “randomized controlled trial,” “randomized controlled experiment,” “randomized controlled study,” “RCT,” “randomized control,” “randomized,” and “controlled random.” English search terms included “Autoimmune Thyroiditis,” “Thyroiditis, Autoimmune,” “Hashimoto thyroiditis,” “HT,” “AIT,” “Se,” “Selenium,” and “Randomized Controlled Trial.”

### 2.2. Inclusion and exclusion criteria

The inclusion criteria for studies: Adults (≥18 years) diagnosed HT with or without hyperthyroidism or hypothyroidism (excluding Graves’ disease). The experimental group will receive Se supplementation alone or in combination with LT_4_, while the control group will receive a placebo or a combination of LT_4_ and a placebo or no treatment. The primary outcome measures will include TPOAb, TgAb, secondary outcomes were thyroid function (TSH, FT_3_, FT_4_) and any adverse effects experienced by the participants. The study design will be either RCTs or prospective studies.

The following exclusion criteria will apply to the literature search: Duplicate literature. Systematic reviews, reviews, meta-analyses, case reports, and meeting records. Low-quality literature or the full text cannot be obtained. Combination with other systemic diseases. The experimental group and the control group took drugs that may affect the concentration of thyroid hormones, autoantibodies, or Se, such as glucocorticoids, traditional Chinese medicine prescriptions, etc. The subjects of the study were pregnant or lactating women. A history of thyroid surgery, previous I^131^ treatment, and a history of using Se preparations within half a year. Animal experiments.

### 2.3. Data extraction

Three reviewers, Heng Zhang, Yunkai Yang, and Shaohua Liu independently reviewed the titles and abstracts of all identified articles and excluded those that did not meet the inclusion criteria for this study. The full text of the remaining literatures was then examined thoroughly and extract the characteristic of included studies. Any discrepancies in opinion were resolved through discussion and consensus-building or by involving an independent third reviewer as a mediator. The information gathered included: the first author, title of the literature, year of publication, and publication journal. The research subjects’ baseline comparability, age, gender, sample size, study design, recruitment period, and study duration. Outcome indicators, including TPOAb, TgAb, thyroid function (TSH, FT_3_, FT_4_), and adverse effects. In cases where the 2 reviewers disagreed, a discussion was held to reach a consensus, or a third party was consulted to make a judgment.

### 2.4. Statistical analysis

We analyzed the data according to the Cochrane Handbook 5.1^[29]^ system evaluation manual, and used Review Manager 5.3 (https://tech.cochrane.org/revman) software to organize and conducted a meta-analysis of the data included in the literature. For all continuous variables, we extracted the data as mean ± standard deviation (SD) and the 95% confidence interval (CI). In cases where the data were initially presented as the median with 95% CI, we calculated the SD using the formula ((HCI − LCI)/2/TINV (0.05; n − 1) × sqrt (*n*)), where HCI and LCI represent the upper and lower limits of the CI, and *n* indicates the sample size of the group. The median was utilized as the mean when data were reported as median with interquartile range (IQR), and the SD was computed by IQR/1.35. The SD was calculated as one-fourth of the range for data presented as the median with range. For binary variable, the relative risk ratio (RR) was calculated. In cases where the measurement units varied between samples, the standardized mean difference was used. Heterogeneity was assessed using both *P*-values and *I*^2^ tests. *I*^2^ was used to quantify the degree of variation in effect sizes among included studies due to heterogeneity rather than sampling error. A fixed effect model was employed when there was no statistically significant heterogeneity among the included studies (*P* > .05 or *I*^2^ < 50%). A random-effects model was used when significant heterogeneity was present (*P* < .05 or *I*^2^ ≥ 50%). Potential sources of heterogeneity were investigated to minimize their impact on the results. When a potential source of heterogeneity was identified, a fixed-effects model was used for meta-analysis. In cases where the source of heterogeneity could not be identified, a random-effects model was used for meta-analysis. Descriptive analysis was used when meta-analysis was not possible (i.e., fewer than 2 studies available), and the final analysis result was considered statistically significant if *P* < .05. Funnel plots were used to assess the possibility of publication bias.

## 3. Results

### 3.1. Retrieval results

**Figure 1.**
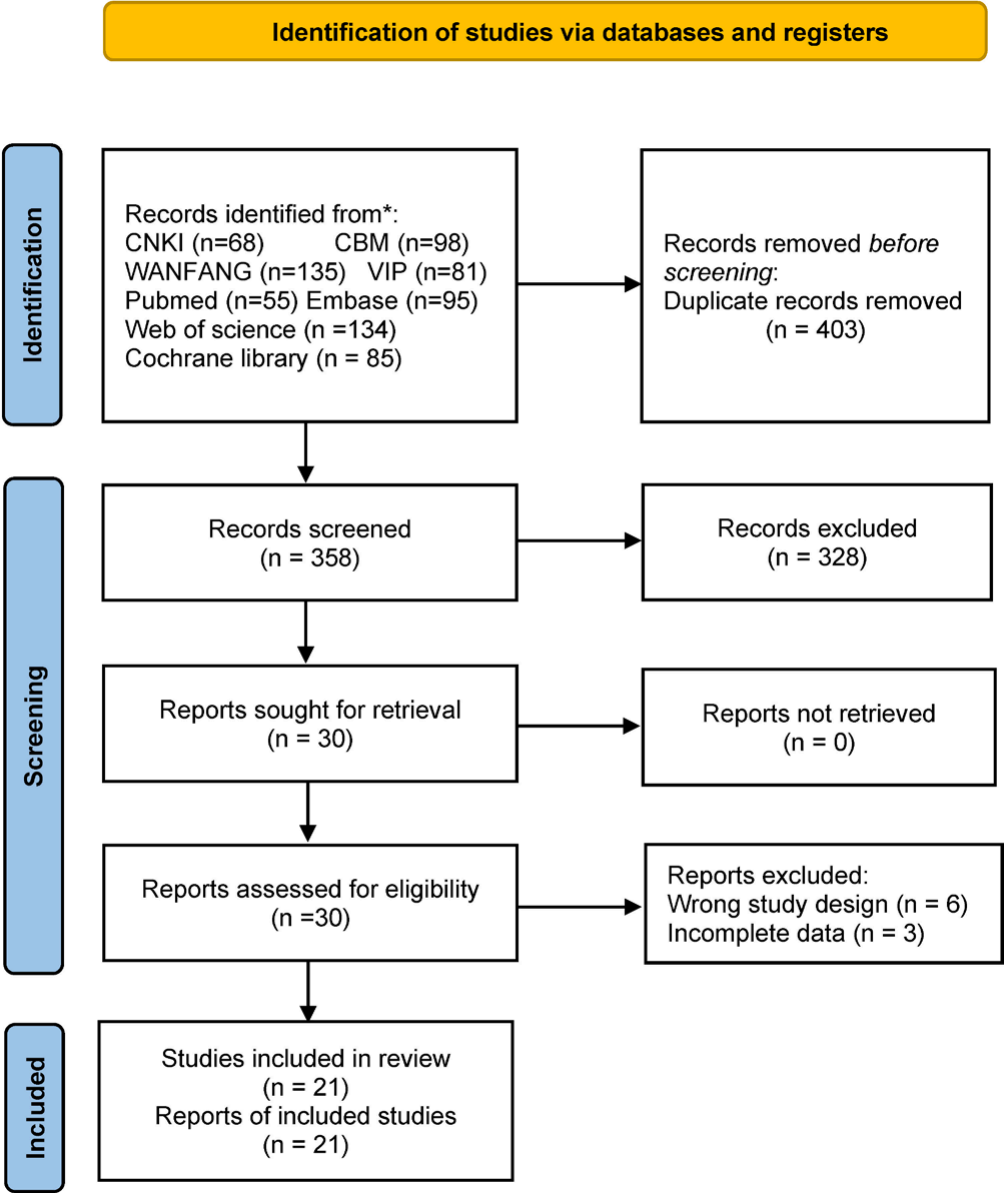
PRISMA flow diagram of study selection. PRISMA = Preferred Reporting Items for Systematic Reviews and Meta-Analyses.

A total of 761 articles were retrieved from 8 databases. After removing duplicates using Endnote 20 software, 358 articles remained. We further screened the titles and abstracts and excluded 328 articles that did not meet the inclusion criteria. Nine additional articles were excluded due to wrongful study design or incomplete data, leaving 21 articles that were carefully reviewed in full text.^[22,23,25,30–47]^ The PRISMA flow chart was used to document the screening process (Fig. 1).

### 3.2. Study characteristics

The characteristics of the included studies are summarized in Table 1. Se levels in the patients included in the studies are recorded in Table S1 (Supplemental Digital Content, https://links.lww.com/MD/P799). We included 21 studies which reported data on 1600 HT patients. One trial lacked randomization,^[42]^ and all trials used a placebo as the control group except 3,^[42–44]^ which used no treatment, and 2,^[30,47]^ which used LT_4_ only. Eight studies were double-blinded,^[31–33,37,39–41,45]^ while one was single-blinded,^[38]^ Three were open label.^[34,36,47]^ Three were described as “blinded” without specification,^[23,25,35]^ and 5 did not account for blinding.^[22,30,42–44]^ Four studies used 2 intervention groups and 2 control groups,^[34,39,40,46]^ while another study had 2 intervention groups and 1 control group.^[38]^ Patients in the intervention group received Se in the form of selenomethionine (Seme),^[22,23,31,32,39,40,42–44]^ selenoyeast (Se-yeast),^[36,46]^ and sodium selenate (NaSe),^[25,30,33–35,37,38,41,45,47]^ all of which were administered orally once daily. The dosage of Se was typically 200μg/d, except for 3 studies with dosages of 1 mg/d,^[45]^ 80μg/d^[43]^ and 83μg/d,^[44]^ respectively. Most studies lasted 3 or 6 months, with 3 studies lasting 12 months.

**Table 1:** Characteristics of included studies in the systematic review and meta-analysis.

Study (First author)	Ref.	Country	Blinded	Sample size (int./con.)	Intervention groups	Age (yr)	LT_4_ treated	Female (%)	Duration (mo)
Wang QH 2017	^[45]^	China	Double	79 (44/35)	NaSe (1mg/d) vs placebo	Mean	Part	84	24 wk
Yu 2017	^[47]^	China	Open label	60 (34/26)	NaSe (200μg/d) vs LT_4_	Mean 37	All	93	3
Turker 2006	^[23]^	Turkey	No specification	88 (48/40)	Seme (200μg/d) vs placebo	Mean 40	All	100	3
Duntas 2003	^[22]^	Greece	Unclear	65 (34/31)	Seme (200μg/d) + LT_4_ vs placebo + LT_4_	Mean 48	All	86	6
Karanikas 2008	^[25]^	Austria	No specification	36 (18/18)	NaSe (200μg/d) vs placebo	Mean 47	All	100	3
Hu 2021	^[36]^	China	Open label	90 (43/47)	Se-yeast (200μg/d) vs placebo	Mean 39	Part	89	6
De Farias 2015	^[32]^	Brazil	Double	55 (28/27)	Seme (200μg/d) vs placebo	Median 46	Part	91	3
Karimi 2019*	^[38]^	Iran	Single	102 (38,36/28)	NaSe (200μg/d) vs vitamin C (500mg/d) vs placebo	Mean 40	Part	75	3
Mazokopakis 2007	^[42]^	Greece	Unclear	80 (40/40)	Seme (200μg/d) + LT_4_ vs Seme followed by no treatment	Median 37	Part	100	12
Krysiak 2012†	^[40]^	Poland	Double	149 (38,37/38,36)	Seme (200μg/d) vs Seme + LT_4_ vs LT_4_ vs placebo	Mean 41	None	100	6
Krysiak 2011†	^[39]^	Poland	Double	165 (42,42/41,40)	Seme (200μg/d) vs Seme + LT_4_ vs LT_4_ vs placebo	Mean 39	None	100	6
Gartner 2003‡	^[34]^	Germany	Open label	47 (13,9/14,11)	NaSe–NaSe (200μg/d) vs NaSe-0 vs placebo-NaSe vs placebo-0	Mean 41	All	100	6
Gartner 2002	^[24]^	Germany	No specification	70 (36/34)	NaSe (200μg/d) vs placebo	Mean 42	All	100	3
Kachouei 2018	^[37]^	Iran	Double	70 (35/35)	NaSe (200μg/d) + LT_4_ vs placebo + LT_4_	Mean 45	All	64	3
Eskes 2014	^[33]^	The Netherlands	Double	61 (30/31)	NaSe (200μg/d) vs placebo	Median 44	None	90	6
Yan 2008	^[30]^	China	Unclear	114 (59/54)	NaSe (200μg/d) + LT_4_ vs LT_4_	Mean 42	None	88	3
Pirola 2016	^[44]^	Italy	Unclear	192 (96/96)	Seme (83μg/d) vs no treatment	Mean 48	None	64	4
Nacamulli 2010	^[43]^	Italy	Unclear	76 (46/30)	Seme (80μg/d) vs no treatment	Median 43	None	86	12
Balazs 2008	^[31]^	Hungary	Double	132 (70/62)	Seme (200μg/d) vs placebo	Mean 42	All	100	12
Mahmoudi 2021	^[41]^	Iran	Double	42 (21/21)	NaSe (200μg/d) vs placebo	Mean 42	None	88	8 wk
Wang W 2018	^[46]^	China	Prospective Cohort	364 (153/28 vs 160/23)	Se-yeast (200μg/d) vs Se-yeast (200μg/d) + LT_4_ vs placebo vs placebo + LT_4_	Mean 41	None	100	6

FT_3_ = free triiodothyronine, FT_4_ = free thyroxin, int/con = intervention group/control group, NaSe = sodium selenite, Se = selenium, Seme = selenomethionine, Se-yeast = selenoyeast, TSH = thyroid-stimulating hormone.

* Patients receiving vitamin C were not analysed.

† This trial had 4 intervention groups, and only the intervention groups receiving Seme or placebo were included in the meta-analysis.

‡ This trial was an open-label follow-up study after that reported in the study by Balazs,^[31]^ in which participants followed different intervention regimes.

### 3.3. Basics bias risk assessment

**Figure 2.**
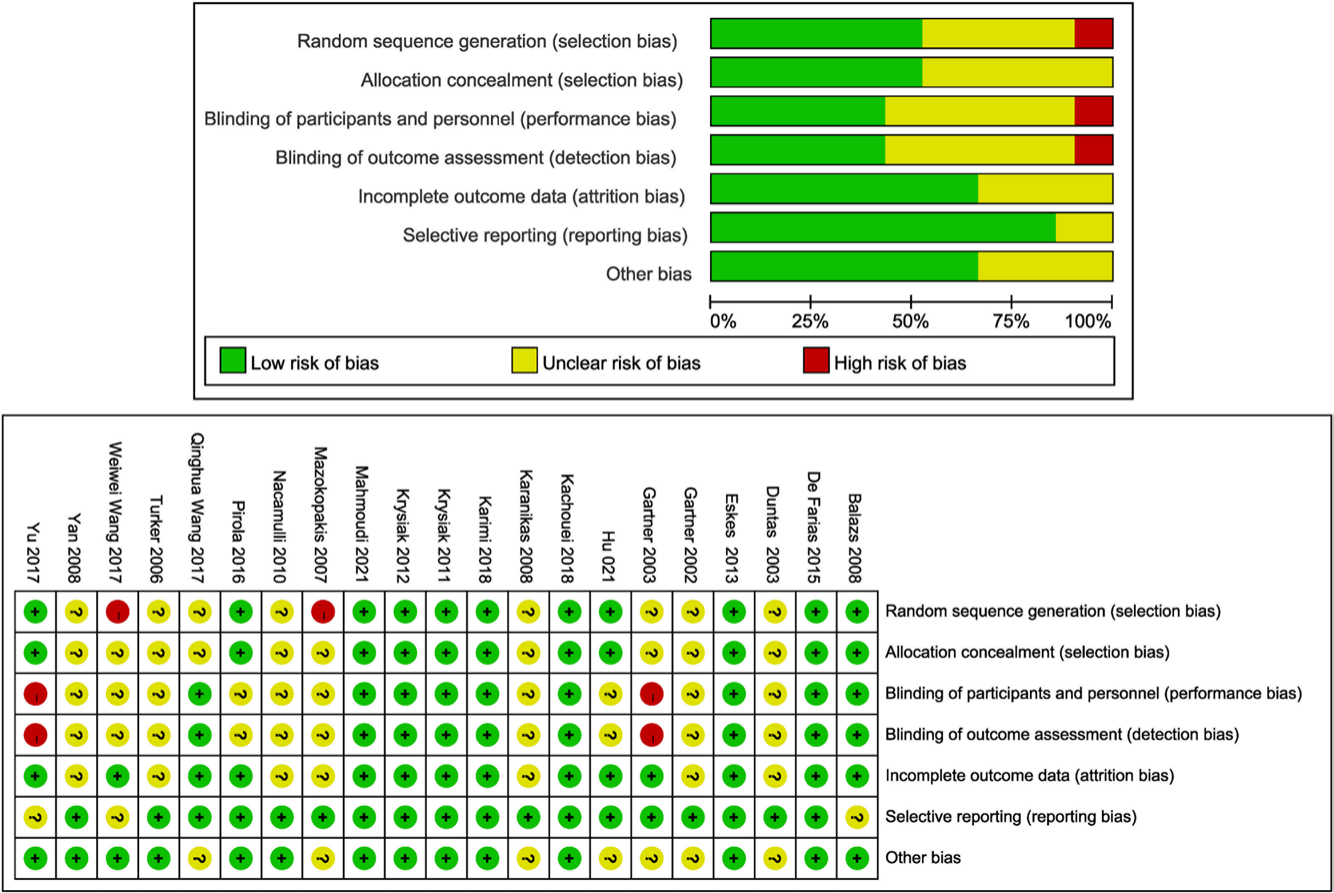
The bias risk evaluation of the included literatures.

The overall quality of each study was evaluated according to the RCT bias risk assessment standard provided by the Cochrane Handbook 6.0. The evaluation criteria included the following: whether the research group used a random method sequence; whether allocation hiding occurred; whether the researcher and subjects were blinded; whether the research result was blinded; whether there were incomplete data; whether selective reporting occurred; and whether there were any other biases. We assessed all included literatures using the evaluation criteria to determine the low-risk, unclear-risk, and high-risk bias of each article. The evaluation of the bias risk is presented in Figure 2.

### 3.4. Systematic review

The meta-analysis found that most of the studies indicated that Se supplementation may lead to a decrease in TPOAb levels, with 3 papers reporting opposite results.^[25,33,41]^ Additionally, Gärtner et al^[35]^ reported that Se supplementation could achieve complete normalization of TPOAb levels and thyroid ultrasound echogenicity in 9 out of 36 patients. In terms of TgAb concentration, the majority of trials did not show a significant change after Se supplementation, while 2 additional trials found a substantial decrease.^[25,36]^ One possible explanation for this is that TgAb is a circulating antigen and not necessarily an antigen specific to thyroid autoimmune responses, which makes it less precise for HT etiology and diagnosis. Regarding changes in TSH levels, statistically significant differences were observed by 2 researchers,^[36,40]^ but no significant differences in TSH were discovered in other studies. One study found that levothyroxine alone or in combination with Seme, but not Seme alone, could lower TSH and raise FT_4_ and FT_3_ levels.^[40]^ In terms of the development of hypothyroidism, Eskes et al^[33]^ reported that 2 of 30 patients in the control group and 2 of 31 patients in the sodium selenite group developed subclinical hypothyroidism during the follow-up period. Karimi and Omrani.^[38]^ reported that 5 patients developed hypothyroidism (TSH ≥ 10 mIU/L), but they were all from the placebo group. Four trials evaluated changes in thyroid ultrasound features. Two studies reported “improved echogenicity” after 3 and 9 months of sodium selenite treatment,^[34,35]^ while 1 trial reported no change in echogenicity or thyroid volume after 3 or 12 months of Se supplementation.^[32]^ The other trial reported increased hypoechogenicity in controls compared to the Se groups.^[43]^ Four studies reported that Se supplementation improved well-being or mood in patients with HT compared to the control group.^[22,25,33,35]^ Two studies using the SF-12 form found that 200μg of sodium selenite per day for 3 months significantly improved patients’ well-being compared to a placebo.^[25,35]^ Eskes et al^[33]^ used the SF-36 form and reported no significant changes in well-being after 6 months of Se supplementation in patients without LT_4_. One trial reported improved mood, sleep, and reduced fatigue in 25 out of 34 participants receiving 200μg/d of Seme for 6 months, with improvements in behavior and tiredness in 15 out of 31 participants in the placebo group.^[22]^ Regarding adverse events, 5 trials reported them. Two studies mentioned gastric discomfort,^[23,42]^ while another study reported side effects such as diarrhea, headaches, and vomiting.^[40]^ Eskes et al^[33]^ reported 2 cases of hair loss, equally distributed in the placebo and Se groups, while Krysiak and Okopien.^[39]^ reported 2 cases with complaints of nausea and headache in the Se group, with no adverse effects in the placebo group.

### 3.5. Meta-analysis

#### 3.5.1. Change in thyroid autoantibody

**Figure 3.**
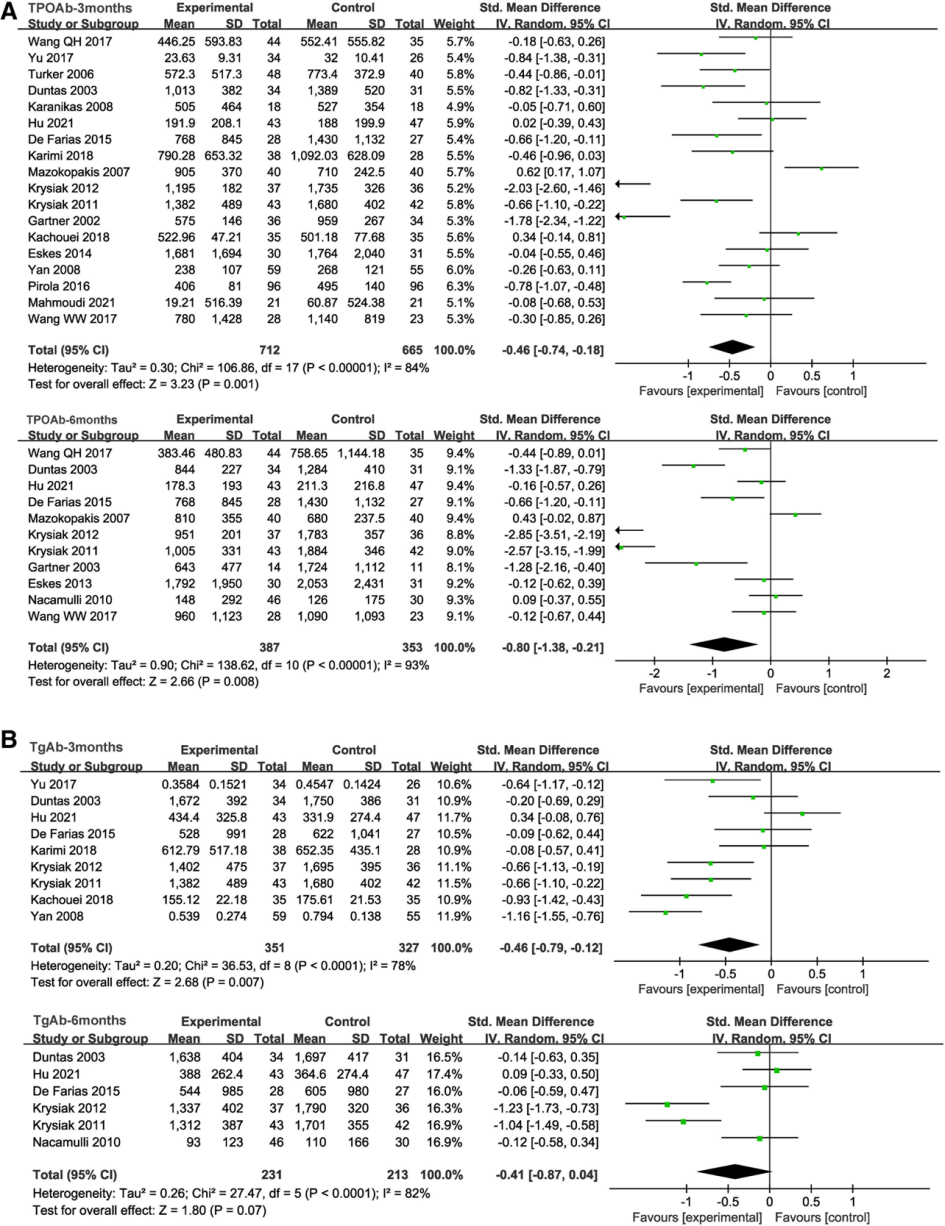
(A) The forest plot comparing the level of TPOAb of patients in the selenium group and the control group after 3 and 6 months. (B) The forest plot comparing the level of TgAb of patients in the selenium group and the control group after 3 and 6 months. TgAb = thyroglobulin antibody, TPOAb = thyroid peroxidase antibody.

Eighteen studies examined changes in TPOAb levels after 3 months and 11 studies examined changes in 6 months. We found that the TPOAb levels of HT patients who received Se supplementation decreased significantly after 3 months (SMD = −0.46, 95% CI: −0.74 to −0.18, *P* = .001) and after 6 months (SMD = −0.80, 95% CI: −1.38 to −0.21, *P* = .008) (Fig. 3A). Nine trials after 3 months of Se treatment showed significant decrease in TgAb levels in Se groups (SMD = −0.46, 95% CI: −0.79 to −0.12, *P* = .007), but no statistically significant difference was found in 6 trials after 6 months (SMD = −0.41, 95% CI: −0.87 to 0.04, *P* = .07) (Fig. 3B).

#### 3.5.2. Change in TSH

**Figure 4.**
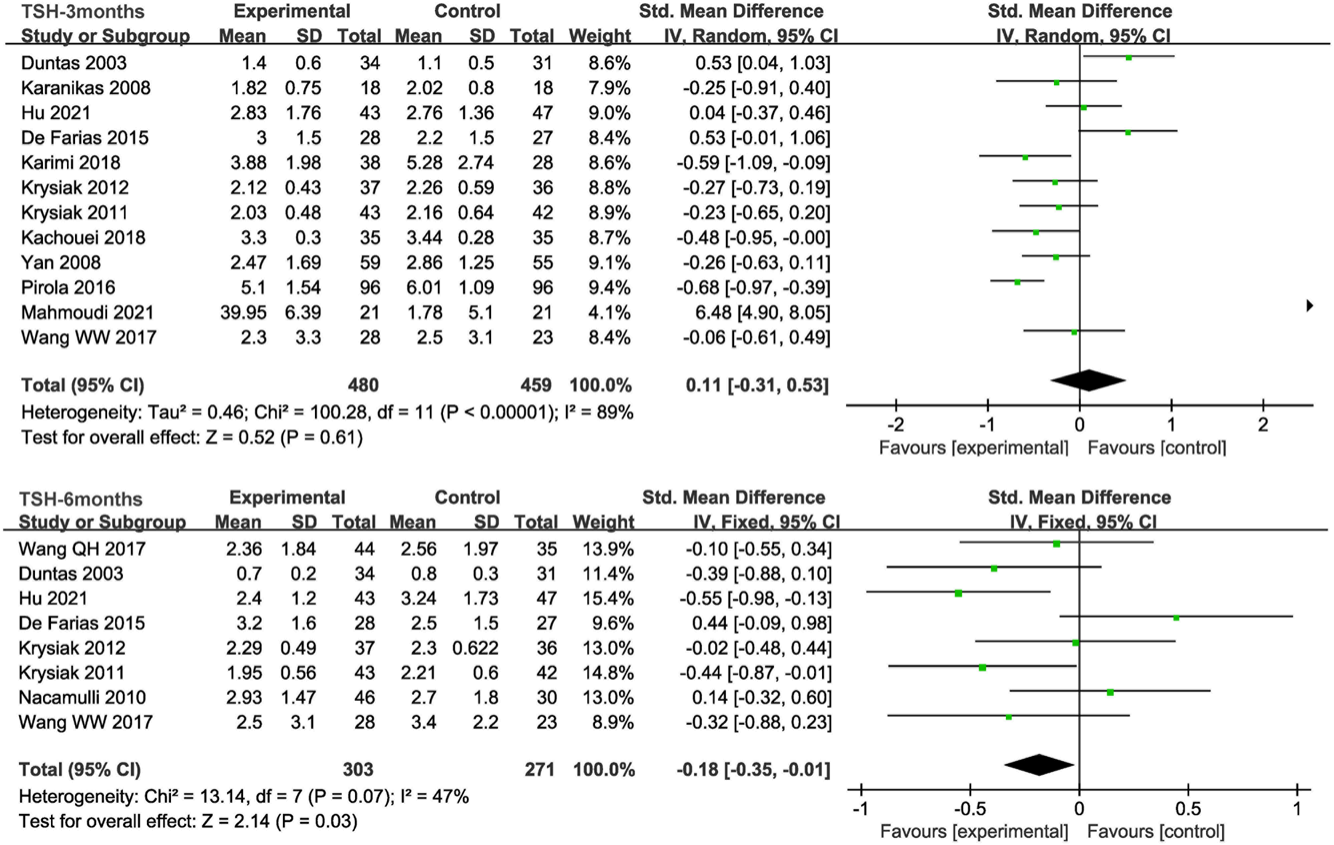
The forest plot comparing the level of TSH of patients in the selenium group and the control group after 3 and 6 months. TSH = thyroid-stimulating hormone.

There was no statistically significant difference in TSH levels between the 2 groups after 3 months from 12 studies (SMD = 0.11, 95% CI = −0.31 to 0.53, *P* = .61). But the analysis results differed based on 8 trials which showed TSH levels significant lowered after 6 months (SMD = −0.18, 95% CI = −0.35 to −0.01, *P* = .03) (Fig. 4).

#### 3.5.3. Subgroup analyses of Se formulations

**Figure 5.**
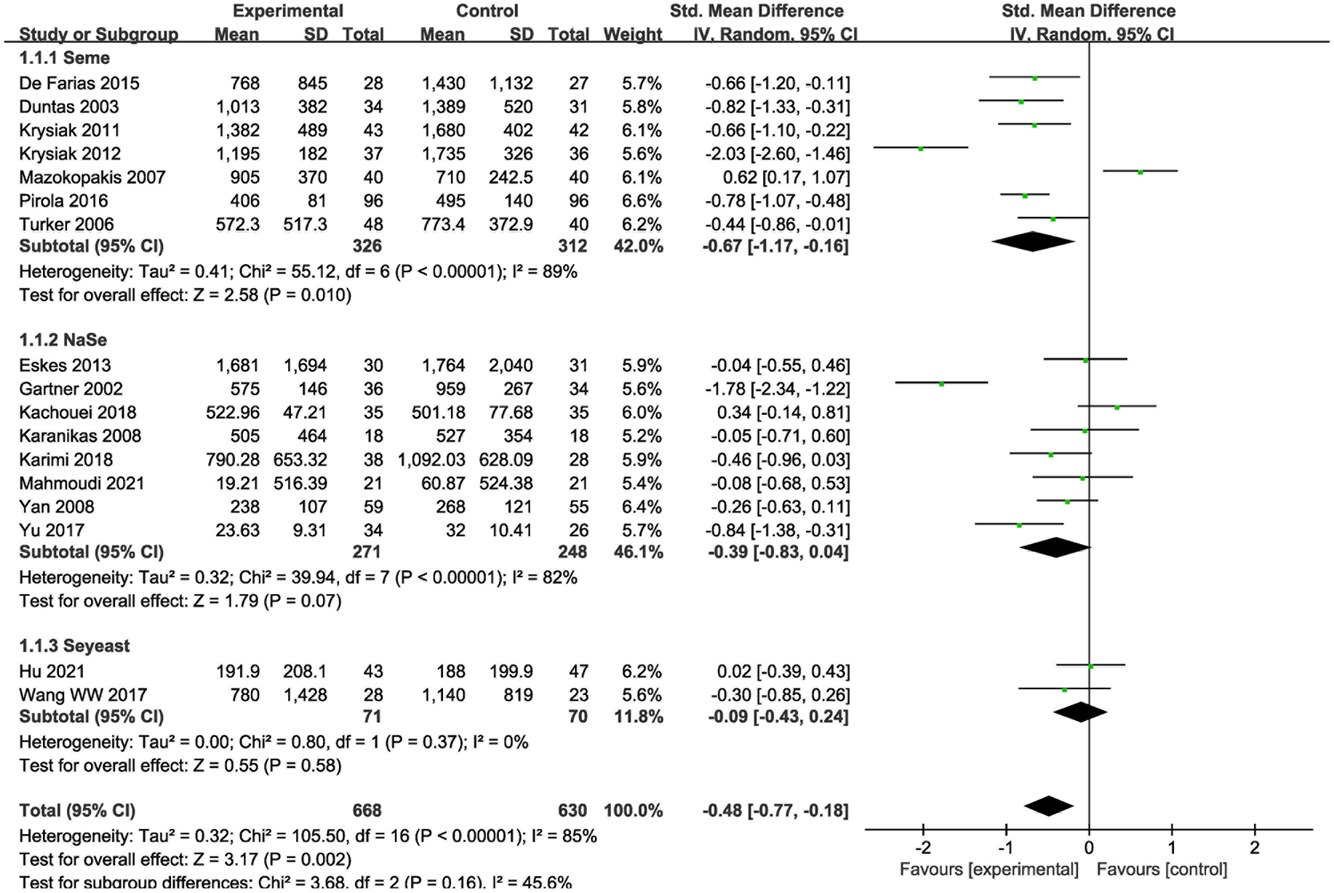
Subgroup analysis based on selenium formulation of TPOAb decrease of patients in the selenium group and the control group. TPOAb = thyroid peroxidase antibody.

The Se formulations used in the studies could be divided into 3 subgroups: Seme, NaSe, and Se-yeast. The Subgroup analyses indicated that 3 months of Seme treatment significantly reduced the TPOAb levels on 7 studies (SMD = −0.67, 95% CI = −1.17 to −0.16, *P* = .01) (Fig. 5).

#### 3.5.4. Change in FT_3_ and FT_4_

The meta-analysis found no statistically significant difference in FT_3_ and FT_4_ level after 3 or 6 months of Se treatment (Fig. S1, Supplemental Digital Content, https://links.lww.com/MD/P797).

#### 3.5.5. Analyses of improvement of well-being

The meta-analysis indicates that participants who received Se supplementation had a significantly higher chance of an improvement in well-being or mood (RR = 2.79, 95% CI = 1.21 to 6.47, *P* = .02) (Fig. S2, Supplemental Digital Content, https://links.lww.com/MD/P797).

#### 3.5.6. Adverse effects

The meta-analysis shows adverse effects were more common in patients who received Se supplementation (RR = 3.90, 95% CI = 1.11 to 13.66, *P* = .03) (Fig. S3, Supplemental Digital Content, https://links.lww.com/MD/P797).

#### 3.5.7. Analysis of publication bias

The funnel plot demonstrated that the markers of TPOAb, TgAb, TSH, FT_3_, and FT_4_ were predominantly concentrated, with very few data points falling outside the funnel pattern. This suggests a low risk of publication bias in the included studies (Fig. S4, Supplemental Digital Content, https://links.lww.com/MD/P797).

## 4. Discussion

This meta-analysis provides strong evidence that Seme is effective in reducing TPOAb titers after 3 and 6 months, as well as TSH titers after 6 months in HT patients compared to control group. Additionally, patients who received Se supplementation had a higher chance of experiencing an improvement in their well-being and/or mood, compared to the control group. Although some patients reported experiencing gastrointestinal discomfort while taking Seme, there were no significant or widespread side effects associated with Se supplementation. These findings suggest that Seme may be a safe and effective treatment option for patients with HT.

Intake levels of Se in population vary around the world due to variations in soil Se content and the impact of plants on Se bioavailability. The human body’s serum Se content follows a “U” curve that corresponds to its physiological effect. Consequently, the ideal range for Se intake is quite limited, and both excessive and insufficient intake of Se will have a detrimental effect on the body.^[13,48]^ Keshan disease was first discovered in China, and it has been conclusively linked to a severe Se deficiency caused by intake of less than 12μg of selenium per day.^[49]^ Additionally, Se deficiency causes thyroid autoimmune illness, Kashin-Beck disease, decreased fertility, and/or decreased reproduction.^[48]^ Similar to other trace minerals, Se is a necessary component of human metabolism but harmful in high doses. The initial indications of Se acute poisoning consist of tachycardia and hypotension.^[50]^ Chronic Se poisoning was first documented in 1961 in portions of the population of Enshi County, Hubei Province, China.^[51]^ It typically presents as alopecia and alterations to the nails. In high-incidence areas, Se intake is predicted to be 4.99 mg on average per day, result in an impact on the skin, the neurological system, and potentially even the teeth. The dietary reference intakes standards for Se are different in countries around the world. The daily Se intake recommended in adults by the Scientific Committee on Food of the European Commission amounts to 55μg/d.^[52]^ According to the World Health Organization, supplementing with Se should not exceed 70μg/d. If the daily consumption of Se surpasses 400 to 700μg, hazardous consequences may arise.^[53]^ It should be underlined that supplementing with Se may only be appropriate for those who are Se deficient, and that determining one’s selenium status is crucial prior to beginning supplementation. Our meta-analysis’ findings also imply that the Se supplementation group experienced adverse effects more frequently than the control group.

Compared with the previous meta-analysis,^[21,54–57]^ we not only added some new studies, but also paid more attention to the effects of different forms of selenium supplementation on HT. We concluded that Selenomethionine is more effective than NaSe and Se-yeast in the teatment of HT.

Theoretically, Se supplementation can protect thyroid tissue and lower thyroid autoantibodies, but in practice, the effects of Se supplementation vary depending on the region and population. The following are the principal opinions of how Se supplementation affects antibodies: Se supplementation can reduce TPOAb and TgAb levels; Se supplementation can reduce TPOAb levels only; Se supplementation does not reduce TPOAb and TgAb levels. This is due to the fact that the concentration of Se in various nations and areas varies, and thus, so does the concentration of Se in the human body. For instance, China has a very unequal distribution of Se resources, and 16 provinces are part of a zone of Se deficit,^[58]^ while the Se level is higher in Venezuela, the United States, Canada and Japan.^[59]^ The level of basic serum Se and the dosage of supplement therapy may have an impact on the effectiveness of Se therapy since the degree of Se insufficiency in HT patients varies, as does the amount of supplement administered.

Based on our systematic review and meta-analysis, we found that Se supplementation in the form of Seme, taken once daily at a dose of 200μg, is effective in lowering TPOAb titers in patients with HT when compared to a placebo. Our findings were consistent at both the 3-month and 6-month follow-up points. The study conducted by Karanikas et al suggested that the different levels of TPOAb titers may represent different immunological states, as they are correlated with T-lymphocyte cytokine production patterns.^[60]^ Additionally, our analysis of data from 2 trials indicated that the response to Se supplementation was dependent on the baseline TPOAb titer, with higher titers being associated with a greater response.^[23,35]^ The observed differences may be attributed to various factors such as variations in the nutritional supply of Se and iodine, differences in patient and regional characteristics, as well as variations in TPOAb titers.^[61,62]^ However, it is worth noting that iodine status was not determined in many of the studies. Many people believe that thyroid antibody level is related to thyroid follicular injury. In fact, thyroid follicular injury is mediated by cells, so the reduction of thyroid antibody level cannot be considered as the reduction of thyroid injury or inflammation, and should be comprehensively analyzed in combination with the indicators of thyroid function.

Our meta-analysis found that Se was effective in reducing TgAb titers within 3 months of treatment, but not after 6 months. It is important to note that TgAb is a general antibody, while TPOAb is a specific marker antibody, which may account for the difference in efficacy observed. Furthermore, the randomization of 2 studies based on baseline TPOAb levels resulted in different baseline TgAb levels between the intervention and control groups. If these studies had been included, our conclusions may have been different. Most of the included studies used TPOAb as the primary indicator, with the other indicators serving as secondary outcome factors. In some studies, TPOAb was the only outcome variable.

Assessing thyroid function is typically done using markers such as TSH, FT_3_, and FT_4_. In patients with HT, hypothyroidism is a common complication. The meta results show the differences was significant at 6 months but not at 3 months. It seem to indicate that selenium supplementation can lead to a decrease in serum TSH levels at 6 months, suggesting that it may be beneficial in improving hypothyroidism. Additionally, the analysis of patient well-being in HT patients supports this finding. However, it should be noted that at 6 months, the difference in TSH level between the treatment group and the control group was very narrow with the CI went from −0.01 to −0.35. Piticchio et al^[63]^ demonstrated that TSH levels are also influenced by the systemic inflammation. Thus it could be explain for the heterogeneity of results. The lack of significant changes in FT_3_ and FT_4_ levels may be due to the fact that TSH is more sensitive and can detect changes in thyroid gland function at an earlier stage than FT_3_ and FT_4_. It is possible that supplementation with selenium may take longer than the observation periods of the included studies (which were typically 3 to 6 months) to produce a significant effect on FT_3_ and FT_4_ levels, perhaps up to 12 months or more. There may need to be additional research that include thyroid function as the primary indicator in order to provide more compelling data, as the majority of the studies included in this analysis did not use it as the primary indicator of observation. It is noteworthy that selenium supplementation is not advised in worldwide guidelines on hypothyroidism.

Research indicates that some dietary patterns influence thyroid function and thyroid antibodies. For instance, following a gluten-free diet may improve selenium absorption, which in turn supports thyroid health.^[64,65]^ Given that TPOAb titers have been shown to be inversely correlated with vitamin D levels, gluten-free diet may also lower TPOAb by increasing vitamin D absorption.^[66]^

In order to assess the impact of a gluten-free diet on thyroid function in patients with HT, Piticchio et al included 4 studies for meta-analysis and reached a relatively cautious conclusion, the findings seem to show that the gluten-free diet has a favorable influence on thyroid function and inflammation as well as an overall trend of decreasing antibody levels, particularly in patients with gluten-related conditions.^[67]^ Due to the small number of included studies, the current evidence is insufficient to recommend this dietary pattern for HT and non-celiac patients, and RCTs in large patient cohorts are still urgently needed.

In our meta-analysis, we conducted a subgroup analysis of the decrease in TPOAb after 3 months according to the Se formulations used in the studies. Seven studies used Seme, 9 studies used sodium selenite, and 2 studies used Se-yeast. The results showed that the Seme subgroup had significantly lower TPOAb levels compared to those receiving sodium selenite and Se-yeast after 3 months. Furthermore, greater changes in autoantibody titers were observed with Seme than with sodium selenite or Se-yeast at the same dose of 200μg. The results seem suggest that the only formulation with significant effects is the Seme. The differences between Se formulations could be explained by the dose–response relationship, as previous studies have reported that the amount of absorbed selenite is roughly two-thirds of that of Seme.^[68]^ This suggests that 133μg of Seme would be equivalent to 200μg of sodium selenite. Se is mainly used to increase the biosynthesis of selenoprotein, and when selenoprotein P and GPx are saturated, only Seme will continue to increase serum Se through its uncontrolled incorporation into various proteins in place of methionine. In contrast, selenite will no longer be effectively used for the biosynthesis of selenoproteins containing selenocysteine and will instead be excreted as selenosugars or methylated Se forms.^[33]^ In other words, when Seme is chosen, the blood Se concentration rises even in well-supplied individuals, based on this, patients with long-term selenium supplementation should regularly monitor the serum selenium level and be alert to the occurrence of selenium poisoning.

Whereas the impact of increased intake of an inorganic Se form (such as selenite or selenite) depends on the subject’s Se status. This explains the superiority of Seme over sodium selenite in reducing TPOAb titers in patients with HT.

There are several limitations in our study. Firstly, some of the included papers had a small sample size and did not provide detailed information on the allocation concealment and blinding methods used in the random-effects model applied in the study, and other analytic results were unclear. This could have impacted the quality of evidence in our study. Secondly, in calculating the mean and SD for the meta-analysis, studies that reported data as medians with interquartile range (IQR)^[36]^ and medians with range^[42]^ may have been less reliable. Lastly, the variability in the techniques used to determine thyroid autoantibody levels in the included studies is a significant weakness of this meta-analysis, as different assays for measuring antibodies may capture a combination of several serum components.^[69]^

Future studies investigating the efficacy of Se supplementation in HT should place greater emphasis on several key factors. Firstly, the Se status of the study population should be clearly established, and clinically important outcomes related to the eligibility criteria for trial participants should be identified. Secondly, to determine which patients would benefit most from Se supplementation, future studies should take into account factors such as response to treatment, years since first diagnosis, and baseline TPOAb titers. Finally, it is important to assess the efficacy of Se in preventing or delaying the progression and deterioration of HT, from euthyroid HT to subclinical and clinical HT. By addressing these key factors, future studies can provide valuable insights into the potential benefits of Se supplementation for individuals with HT.

## 5. Conclusion

Se supplementation help reduce TPOAb and TSH levels in HT patients, leading to improvements in well-being or mood. Selenomethionine is more effective than NaSe and Se-yeast in the teatment of HT.

## Acknowledgments

The author wishes to express their gratitude to the team for their valuable contributions, help, and insightful discussions throughout the project.

## Author contributions

**Data curation:** Yunkai Yang.

**Methodology:** Heng Zhang, Yunkai Yang, Shaohua Liu.

**Writing – original draft:** Heng Zhang.

**Writing – review & editing:** Yang Yang, Zhelong Liu.

## Supplementary Material


