# Supplementary material for: Clinical efficacy of selenium supplementation in patients with Hashimoto thyroiditis: A systematic review and meta-analysis
Source: Medicine (Baltimore). 2025 Aug 29;104(35):e44043. doi: 10.1097/MD.0000000000044043 (PMC12401265; doi:10.1097/MD.0000000000044043)

**Supplementary Figure 1.** The forest plot comparing the level of FT_3_ and FT_4_ of patients in the selenium group and the control group after three and six months.


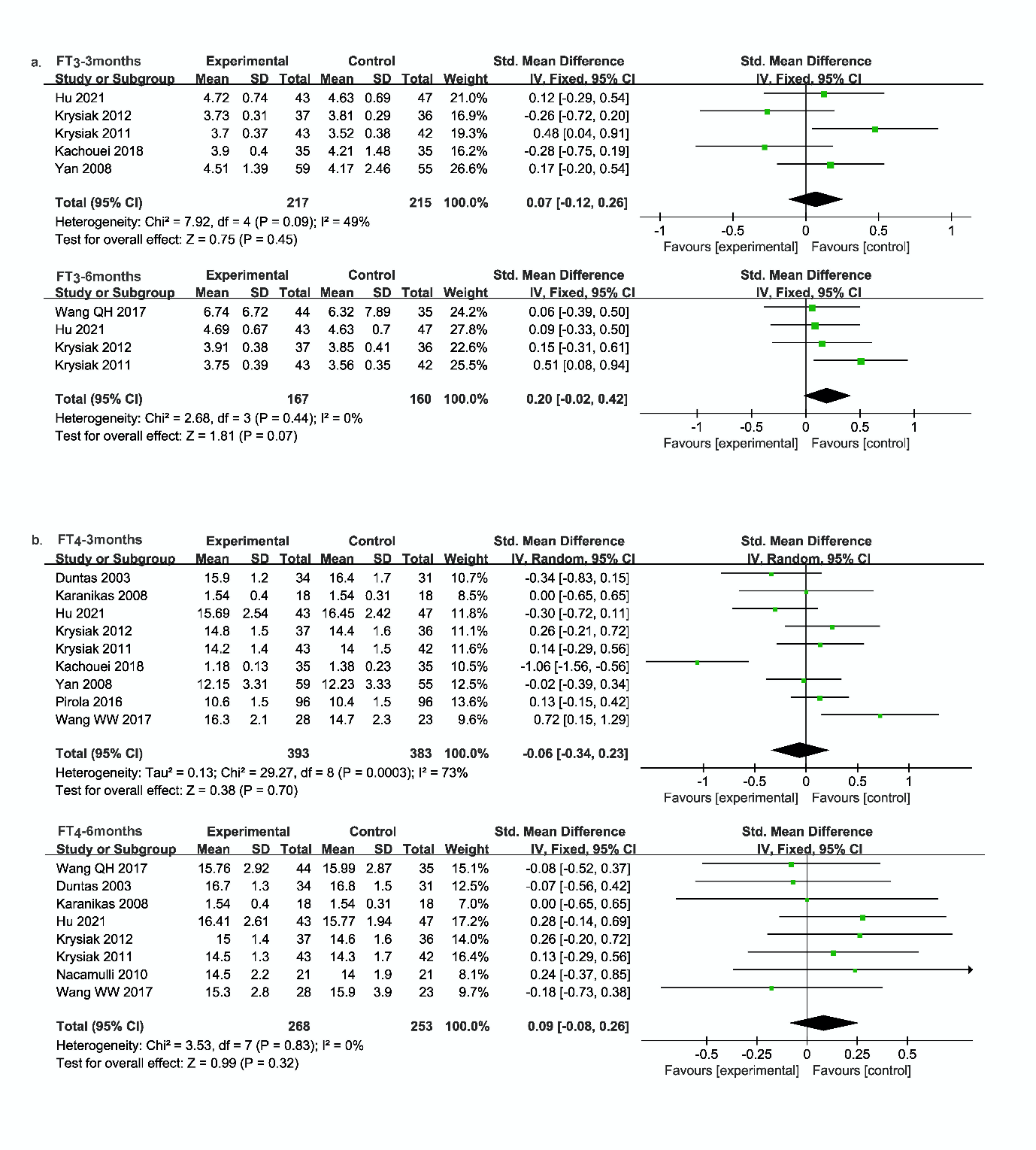
**Supplementary Figure 2.** Analyses of well-being improvement of patients in the selenium group and the control group.


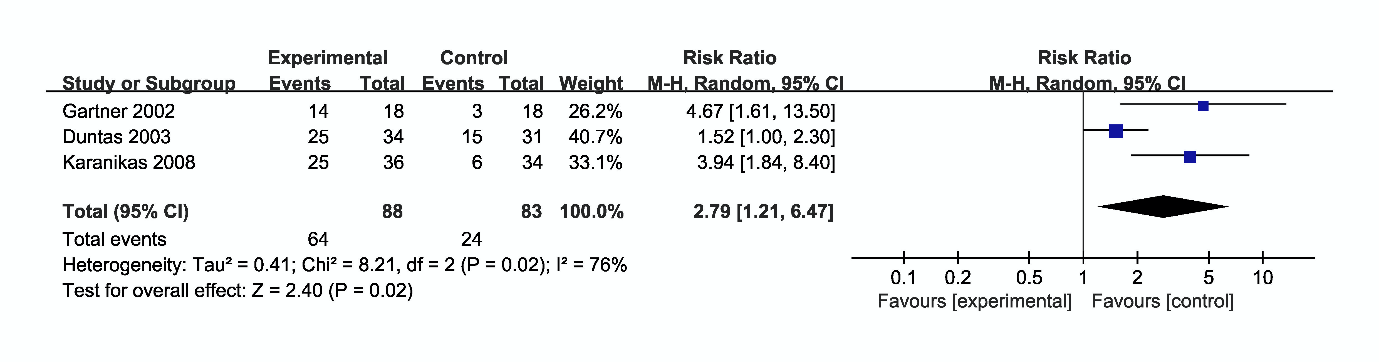


**Supplementary Figure 3.** The forest plot comparing the adverse effects of patients in the selenium group and the control group.


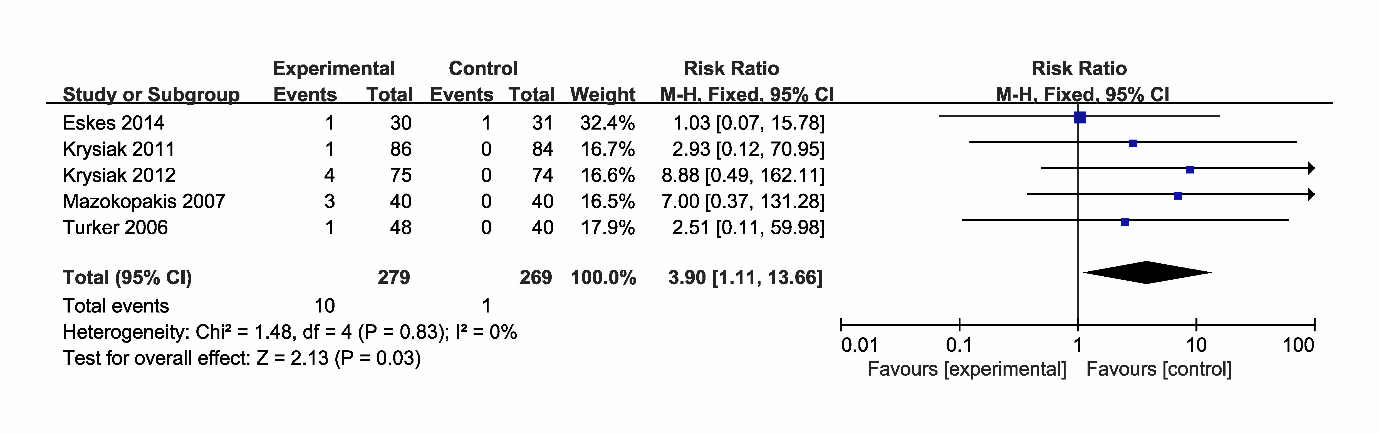


**Supplementary Figure 4.** The funnel charts showing various evaluation indicators.


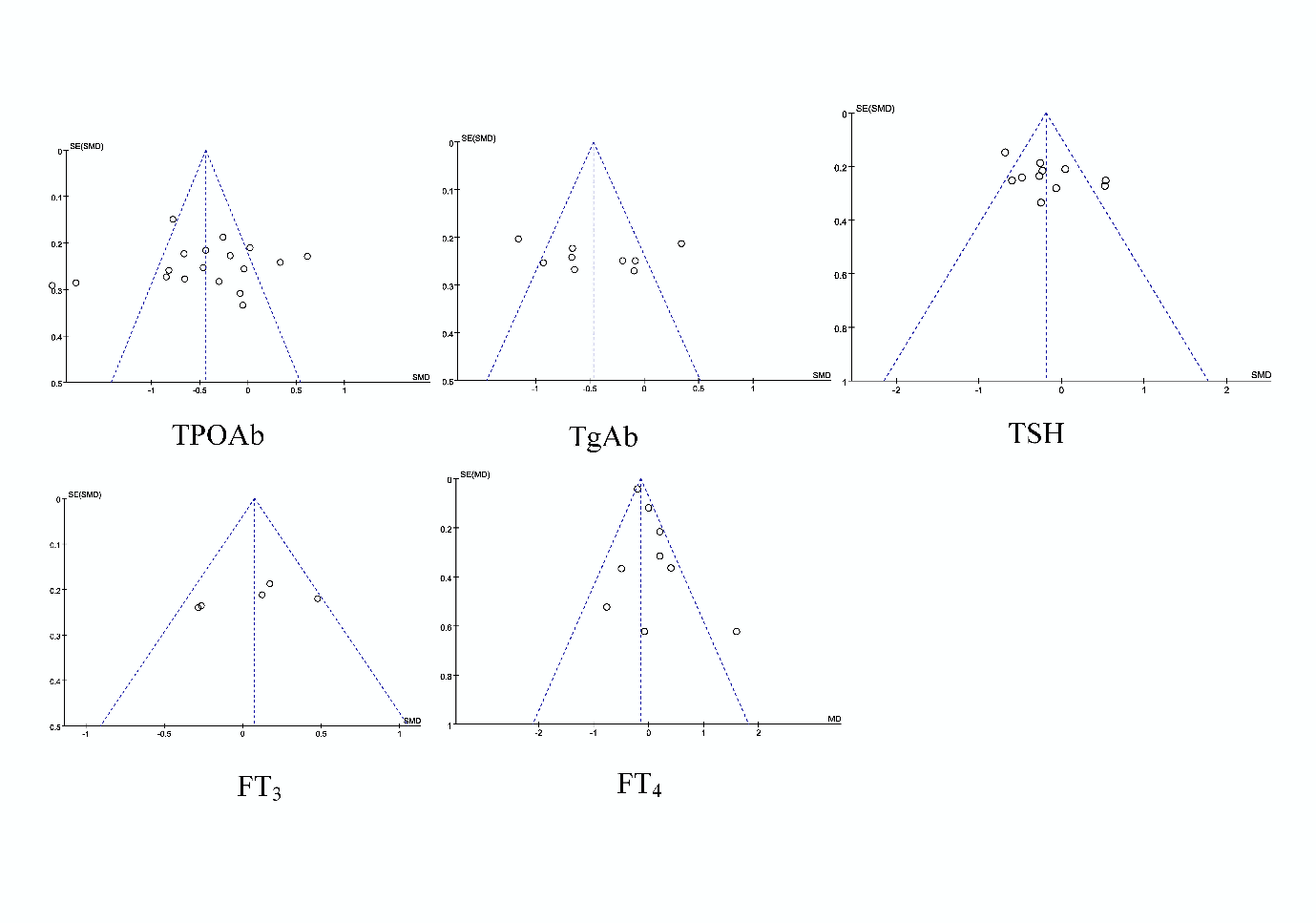

Supplement: Supplementary file 2 [file medi-104-e44043-s002.docx]
